# Fractal complexity of daily physical activity and cognitive function in a midlife cohort

**DOI:** 10.1038/s41598-023-47200-x

**Published:** 2023-11-20

**Authors:** Joanna M. Blodgett, Matthew Ahmadi, Emmanuel Stamatakis, Kenneth Rockwood, Mark Hamer

**Affiliations:** 1 Institute of Sport Exercise and Health, Division of Surgery and Interventional Science, University College London, London, UK; 2 School of Health Sciences, Faculty of Medicine and Health, The University of Sydney, Sydney, NSW Australia; 3 Mackenzie Wearables Research Hub, Charles Perkins Centre, University of Sydney, Sydney, NSW Australia; 4 Geriatric Medicine Research, Department of Medicine, Dalhousie University, Halifax, NS Canada

**Keywords:** Neurology, Risk factors

## Abstract

High stability of fluctuation in physiological patterns across fixed time periods suggest healthy fractal complexity, while greater randomness in fluctuation patterns may indicate underlying disease processes. The importance of fractal stability in mid-life remains unexplored. We quantified fractal regulation patterns in 24-h accelerometer data and examined associations with cognitive function in midlife. Data from 5097 individuals (aged 46) from the 1970 British Cohort Study were analyzed. Participants wore thigh-mounted accelerometers for seven days and completed cognitive tests (verbal fluency, memory, processing speed; derived composite z-score). Detrended fluctuation analysis (DFA) was used to examine temporal correlations of acceleration magnitude across 25 time scales (range: 1 min–10 h). Linear regression examined associations between DFA scaling exponents (DFAe) and each standardised cognitive outcome. DFAe was normally distributed (mean ± SD: 0.90 ± 0.06; range: 0.72–1.25). In males, a 0.10 increase in DFAe was associated with a 0.30 (95% Confidence Interval: 0.14, 0.47) increase in composite cognitive z-score in unadjusted models; associations were strongest for verbal fluency (0.10 [0.04, 0.16]). Associations remained in fully-adjusted models for verbal fluency only (0.06 [0.00, 0.12]). There was no association between DFA and cognition in females. Greater fractal stability in men was associated with better cognitive function. This could indicate mechanisms through which fractal complexity may scale up to and contribute to cognitive clinical endpoints.

## Introduction

Self-affinity, or scale invariance, is a phenomenon where fluctuations across a short period of time resemble fluctuations observed over a longer-period of time^1^. In healthy individuals, self-affinity reflects the successful coordination of the neural and motor systems^2^. In unhealthy states, self-affinity declines and increased randomness is observed^2^. Self-affinity has been observed across various physiological outputs including heart rate^3–5^, respiration rate^6,7^, speech^8^, gait^9–11^ and neural activity^12–15^. Signal data from accelerometers can also provide insight into the complexity of activity fluctuations across the wear period^16^, beyond traditional measures of physical activity volume, intensity or duration^17^. Although acceleration signals from physical activity monitors were initially assumed to solely be due to random noise, exploration of fractal patterns in accelerometer-based data indicates that these fluctuations provide meaningful information into the intrinsic and complex fractal patterns of the individual.

Detrended fluctuation analysis (DFA) captures similarities in fluctuation patterns across time by deriving a scaling exponent to capture consistency of these fluctuations across different temporal scales (e.g. sequential ‘activity windows’ of 1 min, 30 min, 5 h, etc.). Scaling exponents that approach 1 indicate healthy fractal complexity^18,19^, whereas smaller scaling components highlight irregularity in the physiological integration of neural and muscular systems (e.g. 0.5 indicates no correlation). Fractal stability is generally lower in females and declines with increasing age^20^. Lower DFA scaling exponents are associated with higher risk of mortality, frailty and various neurodegenerative outcomes including dementia^20–24^. Early assessment of fractal stability at the individual level may capture physiological dysfunction before it scales up to be clinically visible, and therefore DFA exponents may be a useful biomarker to indicate underlying health dysregulation.

Direct evidence points to poor fractal complexity as an antecedent to cognitive decline^20–24^. Existing evidence suggests strong associations between the disturbance in the fractal complexity of individual motor movements and incidence and progression of dementia^21,23–28^. This is largely thought to be due to underlying neuromotor pathways^28^, where subtle fractal disruptions may be indicative of underlying cognitive impairment. Promisingly, fractal instability, even as an indicator of underlying disease processes, appears to be reversable, with engagement in increased physical activity demonstrating a positive impact in regaining healthy fractal complexity^29–31^. Yet, investigation of DFA scaling exponents in relation to health outcomes has focused solely on older adults, with no investigation in individuals younger than age 50, despite meaningful variation in DFA across the life course^20^. Therefore, our aim was to quantify fractal regulation patterns in 24-h accelerometer data in a midlife cohort at age 46 and examine associations with cognitive function.

## Results

**Figure 1:**
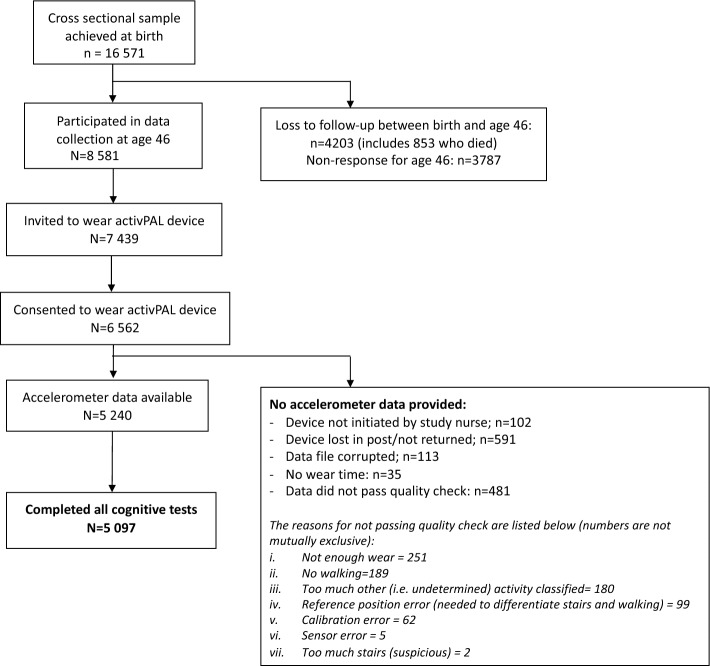
Flowchart indicating the derivation of study sample.

**Figure 2:**
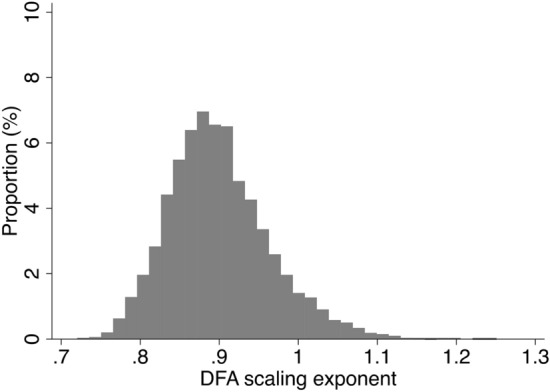
Distribution of detrended fluctuation analysis (DFA) scaling exponent.

The birth cohort sample is described in Table 1, and the derivation of the study sample is provided in Fig. 1. Briefly, participants were aged 46–48 years during assessment, 53.5% (n = 2729) were female, and most of the sample were non-smokers (never smoker: 49.1%, ex-smoker: 32.9%) and either abstinent (10%) or non-problematic drinkers (67.5%). Nearly half of the sample (46%) was educated to A level (typically attained at age 18), with 26% reporting no formal qualifications and 28% having a degree or higher; similarly, nearly half the sample (46.7%) had a lower managerial/ intermediate occupation. Two thirds (65.8%) were married or had a civil partner. Mean body mass index (BMI) was 28.2 ± 5.5 kg/m^2^ and 5% of the sample were classified being severely hampered according to the European Union- Statistics on Income and Living Conditions (EU-SILC) disability definition. Males were more likely to be problem drinkers, have higher BMI, engage in more daily activity and have a professional/managerial occupation. Females were more likely to perform better on the cognitive tests, have formal qualifications, to be married or be widowed/separated, to have a disability and have longer sleep (see Supplementary Table 1). DFA exponents were normally distributed (Fig. 2), with a mean of 0.900 ± 0.646, median of 0.892 (Q1:0.855, Q3: 0.936) and ranged from 0.720 to 1.251.

**Table 1:** Sample characteristics in maximal sample (n = 5097).

	Mean ± SD or n (%)		DFA mean ± SD	
*DFA coefficients*	0.900	± 0.646		
*Cognitive scores*				
Immediate recall: number of words recalled (0–10)	6.7	± 1.4	–	
Delayed recall: number of words recalled (0–10)	5.6	± 1.8	–	
Verbal fluency: number of animals named	24.0	± 6.1	–	
Processing speed: number of letters scanned	346	± 82	–	
Composite cognitive z-score	0.23	± 2.66	–	
*Covariates*				
Sex				
Female	2729	53.5%	0.897	± 0.062
Male	2368	46.5%	0.903	± 0.067
Alcohol consumption (AUDIT-PC group)				
Non-drinker	514	10.1%	0.899	± 0.064
AUDIT score 0–4 (non-problematic drinking)	3432	67.5%	0.899	± 0.065
AUDIT score 5 + (problem drinker)	1136	22.4%	0.900	± 0.064
Smoking status				
I've never smoked cigarettes	2503	49.1%	0.903	± 0.065
I used to smoke but don't at all now	1676	32.9%	0.903	± 0.066
I now smoke occasionally but not daily	235	4.6%	0.897	± 0.065
I smoke cigarettes every day	683	13.4%	0.882	± 0.058
Marital status				
Never married	924	18.5%	0.903	± 0.066
Married or civil partner	3293	65.8%	0.900	± 0.065
Divorced, widowed, separated, former civil partner	788	15.7%	0.895	± 0.061
Highest academic qualification				
No formal qualifications	1313	26.1%	0.896	± 0.063
Up to A levels or diploma (typically attained at age 18)	2313	46.0%	0.897	± 0.063
Degree or higher	1407	28.0%	0.908	± 0.068
Occupational class				
Routine/semi-routine employment	748	16.6%	0.898	± 0.062
Small employer/lower supervisory	807	18.0%	0.889	± 0.060
Lower managerial/ intermediate	2101	46.7%	0.900	± 0.064
High professional/managerial	840	18..7%	0.907	± 0.070
Disability classification EU-SILC				
No EU-SILC long-standing health condition	4308	84.6%	0.900	± 0.065
EU-SILC classification to some extent	533	10.5%	0.896	± 0.061
EU-SILC classification severely hampered	254	5.0%	0.895	± 0.059
Body mass index (kg/m^2^)	28.2	± 5.5	r: − 0.07	
Sleep (hours/day)	6.15	± 1.1	r: 0.04	
Total activity (minutes/day)	151.9	± 51.9	r: 0.10

DFA exponents were slightly higher in males compared to females (0.903 ± 0.067 vs. 0.897 ± 0.062; see Table 1), in non-smokers compared to current smokers, in those with higher levels of educational attainment and those in higher occupational classes. Those who were divorced, widowed or separated had lower DFA exponents, compared to those who were currently or had never married (*p* = 0.10). There was a weak negative correlation between BMI and DFA exponents (r = − 0.07) and weak positive correlation of both sleep (r = 0.04) and total activity with DFA exponents (r = 0.10). There was no difference in DFA exponent amongst disability or alcohol consumption status. Supplementary Table 1 describes covariates and cognitive outcomes by sex.

**Figure 3:**
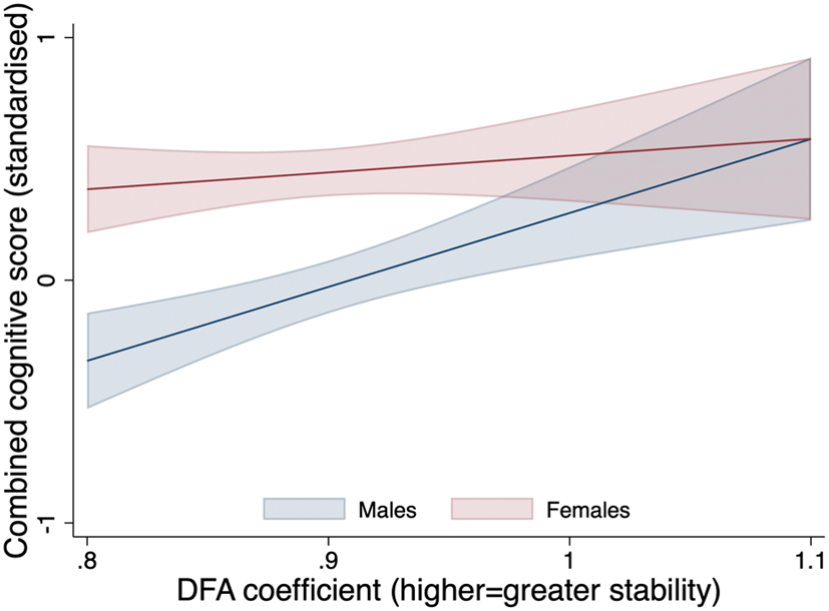
Associations between DFA coefficient and composite cognitive z-score, stratified by females (n = 2729) and males (n = 236).

**Figure 4:**
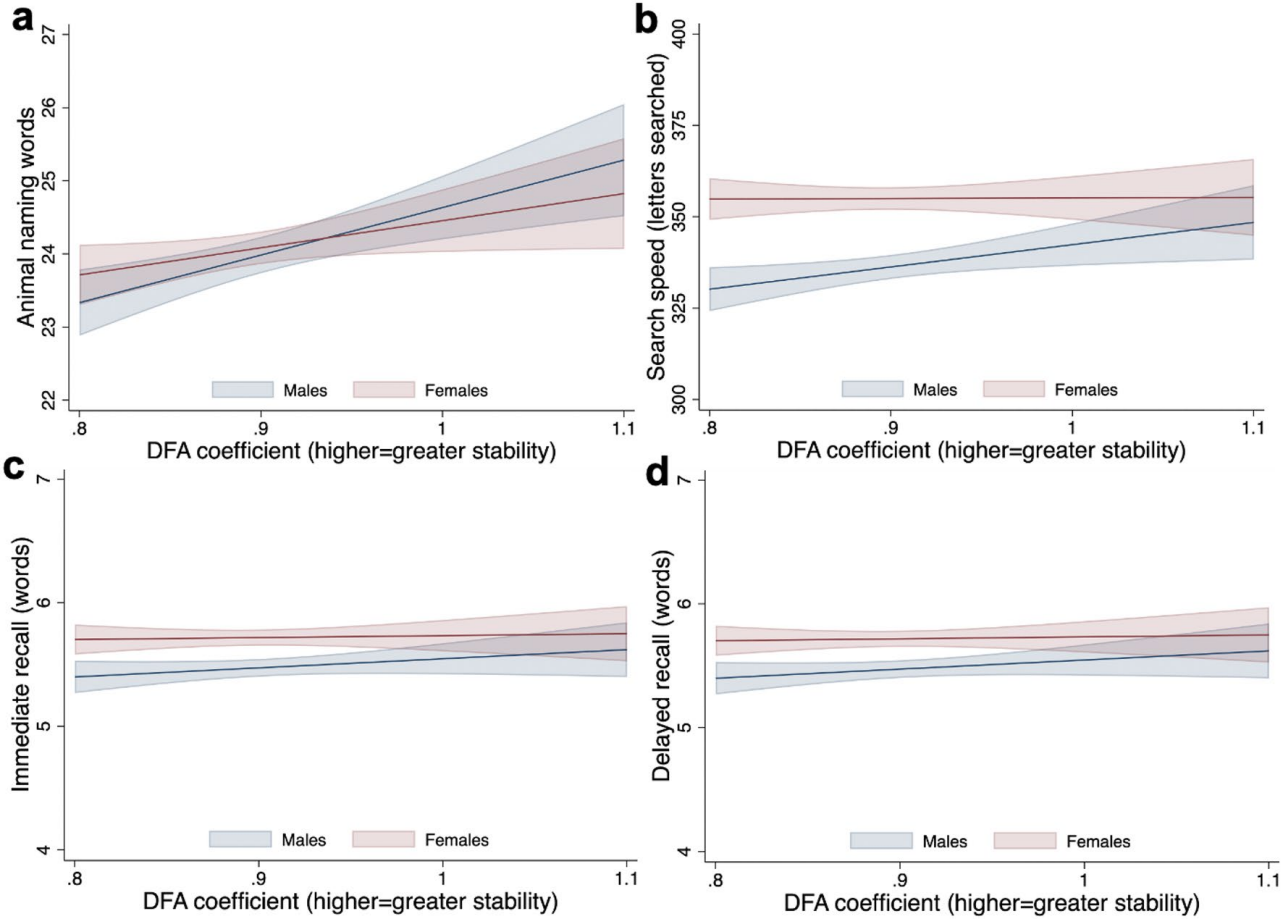
Associations between DFA coefficient and (**a**) verbal fluency; (**b**) immediate recall; (**c**) delayed recall; (**d**) processing speed, stratified by females (n = 2729) and males (n = 2368).

Given clear interactions between sex and DFA (Fig. 3) with composite cognitive z-score, all models were stratified by sex. In females, there was no association between DFA and any cognitive outcome (Table 2, models i–iii). In males, higher DFA was consistently associated with higher cognitive performance. In the unadjusted model, a 0.1 increase in DFA exponent was associated with a 0.30 (95% Confidence Interval: 0.14, 0.47) increase in composite cognitive z-score. The association was attenuated by approximately two thirds after adjustment for education, self-reported health, disability, BMI, smoking, alcohol, sleep, occupational class and total daily PA time (model iii: 0.11 [− 0.05, 0.26]). When examining individual domains, associations were strongest for verbal fluency (Fig. 4). For example, a 0.1 increase in DFA exponent was associated with a 0.10 (0.04, 0.16) and 0.06 (0.00, 0.12) increase in verbal fluency z-scores in unadjusted and fully-adjusted models, respectfully (Table 2; models i and iii). This is equivalent to a 0.65 (0.27, 1.02) and 0.35 (− 0.02, 0.72) increase in the number of animals named (Supplementary Table 2). Although there was some evidence of an association between larger DFA exponent and each of processing speed (0.07 [0.01, 0.13]), immediate recall (0.08 [0.02, 0.14]) and delayed recall (0.05 [− 0.01, 0.11]), associations were fully attenuated in fully-adjusted models.

**Table 2:** Associations between DFA score and each cognitive score at age 46 (β (95% confidence interval); change in z-score per 0.1 increase in DFA coefficient, n = 5 097).

	Model i: unadjusted	Model ii: adjusted for education, self-reported health, disability, BMI, smoking, alcohol, socioeconomic position and sleep	Model iii: adjusted for education, self-reported health, disability, BMI, smoking, alcohol, sleep, socioeconomic position and total PA time
	B coefficient (95% CI)	B coefficient (95% CI)	B coefficient (95% CI)
Females (n = 2729)			
Composite cognitive score	0.07 (− 0.09, 0.23)	− 0.03 (− 0.18, 0.12)	− 0.04 (− 0.19, 0.11)
Verbal fluency	0.05 (− 0.01, 0.11)	0.03 (− 0.03, 0.08)	0.02 (− 0.04, 0.07)
Processing speed	0.00 (− 0.06,0.06)	− 0.00 (− 0.06, 0.05)	− 0.01 (− 0.07, 0.04)
Immediate recall	0.01 (− 0.05, 0.07)	− 0.02 (− 0.08, 0.03)	− 0.02 (− 0.08, 0.03)
Delayed recall	0.01 (− 0.05,0.07)	− 0.03 (− 0.08, 0.03)	− 0.02 (− 0.08, 0.04)
Males (n = 2368)			
Composite cognitive score	0.30 (0.14, 0.47)	0.10 (− 0.05, 0.26)	0.11 (− 0.05, 0.26)
Verbal fluency	0.10 (0.04, 0.16)	0.06 (0.00, 0.12)	0.06 (0.00, 0.12)
Processing speed	0.07 (0.01, 0.13)	0.03 (− 0.02, 0.09)	0.03 (− 0.02, 0.09)
Immediate recall	0.08 (0.02,0.14)	0.01 (− 0.04, 0.07)	0.01 (− 0.04, 0.07)
Delayed recall	0.05 (− 0.01, 0.11)	− 0.01 (− 0.06, 0.05)	0.00 (− 0.06, 0.06)

Missing covariate date imputed using multiple imputation by chained equations.

Cognitive scores were higher in those with valid DFA data (mean composite cognitive z-score: 0.23 ± 2.66) compared to those who participated in the age 46 wave but did not wear an accelerometer (− 0.30 ± 2.74; up to n = 3484). This was true for verbal fluency (animals named: 24.0 ± 6.1 vs. 23.0 ± 6.3), immediate recall (6.7 ± 1.4 vs. 6.4 ± 1.5) and delayed recall (5.6 ± 1.8 vs. 5.3 ± 1.8) but not processing speed (letters searched: 346 ± 82 vs. 347 ± 89). Conversely, the analytical sample had lower DFA exponents (n = 5097, 0.900 ± 0.06) than those missing cognitive data on one or more test (n = 142 with DFA data, 0.912 ± 0.06).

## Discussion

In a large population representative sample of adults in midlife, healthy fractal complexity (i.e. higher DFA scaling exponents indicating stability of fluctuation patterns across different time periods) was associated with better cognitive performance in males. Associations were strongest for executive function measures, with DFA exponent remaining positively associated with verbal fluency independent of socioeconomic measures, health behaviours, health status indicator and total PA levels. There were no associations in females across any cognitive measure. These novel findings indicate that meaningful decline in fractal complexity may begin to emerge well before clinical presentation, possibly decades earlier. Assessment of fractal complexity in midlife may be a clinically promising method to capture meaningful deterioration in neural motor coordination before it becomes clinically visible.

Despite increased recognition of the importance of midlife factors for dementia risk^32,33^, no other study has examined associations between fractal complexity and health outcomes prior to age 50, therefore comparison to existing evidence is limited. However, previous investigation in older adults has demonstrated robust associations between disrupted fractal complexity in motor activity and both the onset and progression of dementia^21,23–28^. In a US cohort study of > 1000 adults with no baseline dementia diagnosis (mean age: 79.7 ± 0.2), lower DFA exponents were strongly associated with increased risk of Alzheimer’s disease diagnosis and a faster decline in global cognitive function^25^. Although five distinct cognitive domains were assessed across 19 tests, authors did not explore how associations differed by cognition type. Further exploration in this cohort indicated that fractal degradation progressively worsened over time, even at pre-clinical stages of Alzheimer’s disease, with an accelerated decline after diagnosis^21^. Other studies have reported similar robust patterns of associations with clinical cognitive end-points^23,24,26–28^. Notably, one study reported opposing sex patterns to those observed above, suggesting that disrupted fractal complexity was associated with amyloid plaque pathology in females but not males^24^. Differing results in the present study may be due to the sample being older (mean age: 65.9 ± 8.3), having insufficient power (n = 61 males) or differences in ascertainment of neuropathological versus cognitive outcomes. The effect sizes presented in this study are smaller than those reported in studies on dementia or mortality outcomes^20,23,24,26–28^, which is not unexpected to the young age of the participant. Nonetheless, the robustness of associations, even after adjustment for key confounders, suggest that this physiological relationship emerges at a younger age than previously expected.

A central hypothesis explaining the link between reduced fractal activity regulation and cognitive impairment focuses on the neuroanatomical changes in the suprachiasmatic nucleus ^34–36^. Degeneration in the suprachiasmatic nucleus, the area of the brain responsible for behavioral and physiological circadian rhythms^34^, may explain why disruptions in fractal activity arise before cognitive impairments become clinically visible^28^. The suprachiasmatic nucleus is located in the anterior part of the hypothalamus, which plays an integral role in coordinating and maintaining homeostasis across many of the body’s systems^37^. Therefore, irregular fluctuations in motor activity might indicate underlying degeneration in this central area. This hypothesis is consistent with patterns from other pre-clinical measures of health. For example, a frailty index constructed of abnormal blood biomarkers captures damage at a subclinical level^38,39^ and can predict adverse health outcomes including mortality even in young, otherwise healthy adults^40^.

Crucially, the presence of this association at age 46 indicates that self-affinity may begin to break down earlier than previously recognized, which is consistent with a life course approach of accumulation of damage at the cellular, organ and system level^41^. This steady decline in DFA scaling has been previously observed in a US cohort of participants from age 6 to 85^20^. The lack of association of DFA scaling exponents with cognitive function in women is surprising, although there was some indication of an association in unadjusted verbal fluency models. We hypothesize that women may be able to withstand a greater degree of poor fractal complexity in midlife, before it impacts clinical outcomes. When considering the emergence of the male–female health-survival paradox in later life^42^, this has important implications. Women often exhibit poorer health than age-matched male counterparts, with greater levels of frailty^43^, healthcare use, disability^44^, cognitive impairment^45,46^, yet often have lower mortality risk and longer life expectation^43,47^. This may be due to a better intrinsic capacity to handle ill health and damage at the cellular and system level, which could explain why women had poorer DFA exponents than men, yet better scores on cognitive tests. Conversely, other hypotheses have suggested that sex difference in lifestyle behaviours, social roles or health-care seeking behaviours may explain why women appear to have poorer health than men^42^—for example, higher disability observed in this study-, yet better survival outcomes.

Associations were strongest for verbal fluency and weakest for both immediate and delayed recall. This was surprising, given the hypothalamus plays a central role in learning and memory^37^—and thus the memory recall tasks-, and reduced fractal activity regulation is hypothesized to result from impairments in the anterior hypothalamus^34–36^. Verbal fluency – measured as the number of animals participants could name within 1 min—may have been a more sensitive and demanding measure of fluid cognition, compared to the cruder 10-word immediate and delayed recall tasks. For example, the animal naming task relies upon several aspects of executive function including clustering and self-initiation (e.g. generating a series of animal names that make up a specific cluster such as bird type, fish type), task switching (e.g. switching to the next cluster after all names in a single species has been exhausted) and action inhibition (e.g. suppressing the tendency to repeat previous names or names that do not fit within the instructions)^48,49^. Therefore, it may more sensitively capture neuroanatomical changes in the hypothalamus.

Key strengths of this study include the large population representative and age-homogenous sample, ascertainment of motor activity using a continuous 7-day wear period with a thigh worn device, and assessment of several cognitive domains with tests that are commonly used in large epidemiological studies^50,51^. Limitations include the cross-sectional nature of the data, loss to follow-up of participants across the life course, and lower cognitive scores in those who did not wear an accelerometer. Due to the cross-sectional design, we are unable to rule out reverse causality, as it is possible that cognitive impairment may cause poor fractal stability given some of the neural mechanisms described above^34–37^. Additionally, there was no assessment of neuropathological outcomes, which could have provided further insight into mechanisms through which fractal complexity may influence cognitive function before impairment manifest clinically.

Our findings need replication in longitudinal studies examining associations throughout midlife to better understand the age (and threshold) at which fractal complexity can meaningfully identify those in need of early intervention. It is also important to better understand the neuropathological mechanisms through which the loss of self-affinity impacts cognitive performance, particularly to understand how it scales up to Alzheimer and dementia outcomes. The significance of this lies in the potential connection between increased exercise and improvements in fractal complexity^29–31^. Should longitudinal evidence reveal a meaningful relationship between fractal complexity and cognitive clinical end-points, increased physical activity may emerge as a promising avenue to influence cognitive health, with fractal complexity as a potential mediator. Another important area of exploration is to investigate how these associations may differ across key characteristics given DFA gradients across most covariates; the role of occupational physical activity may play an intriguing role given known negative effects of occupational physical activity on various health outcomes^52^. Finally, studies that can combine device-assessed physical activity measurements and neuroimaging are needed to examine the pathological characteristics of those who demonstrate poor fractal complexity in midlife. This could provide further insight into the common discordance between underlying neuropathology or genetic risk and clinical expression of cognitive impairment^53,54^.

In summary, we identified robust associations between poor fractal complexity and worse cognitive function in males in midlife. Considering the robust associations of fractal complexity with both the onset and progression of clinical cognitive outcomes (e.g. Alzheimer’s disease)^21,25^, early identification has enormous potential to identify those at early risk. In addition to increasingly accurate, accessible and detailed assessments of physical activity^55^, consumer wearables have seen the implementation of increasingly complex biotechnologies including photoplethysmography and single-lead electrocardiography^56^. Incorporation of fractal physiology into these wearables could one day be feasible by embedding the algorithms used in this study. This holds enormous promise for the future of personalized medicine and early intervention.

## Materials and methods

### Sample

The 1970 British Cohort Study is a birth cohort of over 17,000 individuals born in England, Scotland or Wales during a single week in April 1970^57^; there were no further exclusion criteria. Participants have been followed up to ten times across life, including the most recent data collection at age 46–48 (over an 18-month period, hereafter referred to age 46 wave). At age 46, study members participated in a home visit which consisted of a self-completion questionnaire, a computer-assisted personal interview and a biomedical assessment including a battery of cognitive tests. Participants were subsequently invited to wear a thigh-mounted accelerometer (activPAL3 micro; PAL Technologies Ltd., Glasgow, United Kingdom) for a 7-day wear period. All participants provided informed consent and the most recent ethical approval was given by NRES Committee South East Coast- Brighton & Sussex (ref: 15/LO/1446). This research was conducted in accordance with the ethical standards laid down in the 1964 Declaration of Helsinki and its later amendments. Any individuals who lacked capacity to consent were not included. Of the 8581 individuals who participated in the age 46 wave, 5097 had accelerometer and cognitive data and thus were included in the analytical sample (see Fig. 1 for detail on derivation of sample). Therefore, inclusion criteria were: (i) participated in age 46 data collection wave; (ii) provided valid accelerometer data (minimum of 3 weekdays and 1 weekend of wear time; where a valid day was 10 + h); (iii) had data on all four cognitive tests.

### Device-measured DFA assessment

Participants were fitted with the activPAL3 micro device (frequency: 20 Hz) on the midline anterior part of the upper thigh during the home visit^58^. The device was waterproofed with an adhesive and participants were instructed to wear the device continuously for at least seven days, including while sleeping, bathing, swimming or participating in any activity. Participants were also instructed not to re-attach the device if it fell off accidentally or was removed; devices were returned to the research team via post. Partial data from the first day was removed, thus the seven-day collection period commenced at midnight on the day the device was fitted. The raw data acceleration signal from each 1 s window was processed to derive the signal vector magnitude measuring movement acceleration along three planes of movement (x, y, z axes); this yielded up to 60,480 data points per participant (60 s * 60 min * 24 h * 7 days) provided. To be included in analysis, participants must have 3 weekdays and 1 weekend day of wear time, where a valid day was 10 + h.

To derive the DFA scaling components, standard procedures were followed^59^. First, all non-wear and sleep time were removed from the continuous wear data. Twenty-five distinct time series window sizes were selected (range: 30 s–10 h); note the log of this range was taken to ensure non-linear equally spaced window sizes. Each acceleration signal value was mean centred, by subtracting the average acceleration signal across all valid wear time from each data point. Next, the signal was divided into non-overlapping windows of the chosen size (e.g. consecutive 30 s windows across the whole time series). A least squares line was fitted to the data in each window; for example, in the 10-h time series, a single slope value was produced for each 10 h window representing the trend in that window. The signal was ‘detrended’ by taking the root mean square fluctuation of the residuals from each window. This was repeated across all 25 window sizes. Finally, the log of the root mean square residual values was plotted against the log of the window size; the slope of this line gives the DFA scaling component. A linear relationship on a log–log plots suggests a power law relationship (i.e. fractal scaling)^60^. Therefore, a DFA scaling exponent of ~ 0.50 indicates no correlation in the fluctuations, while values > 0.5 indicate positive correlations, with increasing fractal complexity as DFA exponent surpasses 1.0^19,59^.

### Cognitive outcomes

Four cognitive tests were administered at age 46 to measure (1) immediate short-term memory (word memory); (2) verbal fluency (animal naming); (3) processing speed (letter search) and (4) delayed-recall (delayed word memory). These cognitive assessments have been used in many large-scale epidemiological studies and have been validated for use in population representative samples as sensitive measures of executive function, memory and are established predictors of increased dementia risk^61–67^.

First, participants were verbally presented with 10 words at a rate of one word every two seconds. They were then asked to immediately state all the words that they could remember. *Immediate recall score* was the total number of words they correctly recalled within two minutes. Next, *verbal fluency* was assessed using a task in which participants named as many animals as they could within one minute. Different breeds (e.g. dog, terrier, poodle) or gender/generation-specific names (e.g. bull, cow, calf) counted as distinct names, while redundancies (e.g. brown cow, black cow) and specific characters (e.g. Bambi) did not. *Processing speed* was assessed using a visual search speed task. Individuals were given a grid of numbers and were instructed to go line by line and cross our all the ‘P’s and ‘W’s as quickly and as accurately as possible in one minute. The score was the total number of letters searched. Finally, individuals were asked to verbally recall the ten words from the immediate recall task; *delayed recall score* was the total number of correct words they recalled within two minutes. *A combined standardised cognitive score* was derived by summing the z-scores of each individual task^50^.

### Covariates

Covariates were a priori chosen based on established associations with fractal complexity and cognition^21,22,25,68^. *Sex* was coded as female or male. *Current legal marital status* was based on self-reported data at age 46 and existing information from previous sweeps and was categorized as ‘never married’, ‘married or civil partner’, and ‘divorced, widowed, separated or former civil partner’. *Education* was ascertained as the highest completed academic qualification recorded across any data collection sweep and categorized as: ‘none’, ‘up to A levels or diploma (typically attained at age 18)’ or ‘degree or higher. *Employment types* were coded using the National Statistics Socioeconomic Classifications of Occupations^21^ as: routine/Semi-routine employment, small employer/lower supervisory, lower managerial/ intermediate, high professional/managerial.

*Smoking status* was categorized as ‘never smoker’, ‘previous smoker’, ’occasional smoker (not daily)’ and ‘daily smoker’. *Drinking status* was categorized as ‘non-drinker’, ‘non-problem drinker’ or ‘problem drinker’, derived from five-item the Alcohol Use Disorders Identification Test^69^. *BMI *(kg/m^2^) was derived from study-nurse assessments of height and weight; where missing, self-reported height and weight values were used. Based on a series of questions about longstanding physical or mental health conditions or illnesses lasting or expected to last for 12 months or more, *disability* was categorized as ‘none’, ‘limited to some extent’ and severely hampered’ using the EU Statistics on Income and Living Conditions classification of disability. Finally, *average daily total PA time* (any ambulatory movement), *sleep and wear time* were determined using the validated ActiPASS software, which applies a decision tree model to classify activities based on the signal standard deviation and tilt angle^70,71^.

### Statistical analyses

Descriptive characteristics provided mean ± SD and frequency (proportion) across all covariates and cognitive outcomes. We assessed normality of DFA scores and each cognitive outcome using Kolmogorov–Smirnov tests^72^. One-way ANOVAs and Pearson correlations were used to assess associations between DFA scores and all covariates. Chi-square tests and one-way ANOVAs were used to assess differences in covariates and cognitive outcomes between females and males. Interactions between sex and DFA coefficient were assessed, whereby analyses were stratified by sex if *p* < 0.05. We used linear regression to examine associations between DFA coefficients and each cognitive outcome in three models: (i) sex-adjusted (or stratified) models, (ii) model i + education, self-reported health, disability, BMI, smoking, alcohol and sleep-adjusted models and (iii) model ii + additionally adjusted for total PA. Following a missing at random assumption, covariate data were imputed using multiple imputation by chained equations in Stata V.17 (Stata, College Station, TX, USA). Missing data ranged from 0% (smoking, total PA time) to 1.8% (marital status); estimates from 25 imputed datasets were combined using Rubin’s rules^73^. Characteristics of individuals missing cognitive or DFA data were described and compared to the analytical sample.

### Significance statement

This novel study demonstrated that increased fractal complexity is associated with worse cognitive function in midlife. To our knowledge, this is the first study to demonstrate that associations emerge before clinical onset of cognitive impairment and highlights the potential of fractal complexity as an early indicator of those at risk of cognitive decline.

### Supplementary Information

Supplementary Table 1.

## Data Availability

The BCS70 datasets are publicly available in the UK Data Archive repository: BCS70 https://discover.ukdataservice.ac.uk/series/?sn=200001.
