# Supplementary material for: Fractal complexity of daily physical activity and cognitive function in a midlife cohort
Source: Sci Rep. 2023 Nov 20;13:20340. doi: 10.1038/s41598-023-47200-x (PMC10663528; doi:10.1038/s41598-023-47200-x)
Supplement: Supplementary file 1 — Supplementary Table 1. [file 41598_2023_47200_MOESM1_ESM.docx]

| **Supplementary Table 1.** Sample characteristics in maximal sample, by males and females n=5097) | | | | | |
| --- | --- | --- | --- | --- | --- |
|  | **Males**  **(n=2,368)** | | **Females**  **(n=2,729)** | | **p-value^a^** |
| **COGNITIVE SCORES, mean ±SD** |  |  |  |  |  |
| Immediate recall: number of words recalled (0-10) | 6.7 | ±1.4 | 6.8 | ±1.4 | <0.005 |
| Delayed recall: number of words recalled (0-10) | 5.5 | ±1.8 | 5.7 | ±1.8 | <0.001 |
| Verbal fluency: number of animals named | 23.9 | ±6.2 | 24.1 | ±6.0 | 0.41 |
| Processing speed: number of letters scanned | 336 | ±82 | 355 | ±82 | <0.001 |
| **COVARIATES, mean ±SD or n(%)** |  |  |  |  |  |
| **Alcohol consumption (AUDIT-PC group)** |  |  |  |  |  |
| Non-drinker | 202 | 8.6% | 312 | 11.5% | <0.001 |
| AUDIT score 0-4 (non-problematic drinking) | 1,442 | 61.0% | 1,990 | 72.2% |  |
| AUDIT score 5+ (problem drinker) | 719 | 30.4% | 417 | 15.3% |  |
| **Smoking status** |  |  |  |  |  |
| I've never smoked cigarettes | 1,140 | 48.1% | 1,363 | 50.0% | 0.47 |
| I used to smoke but don't at all now | 782 | 33.0% | 894 | 32.8% |  |
| I now smoke occasionally but not daily | 114 | 4.8% | 121 | 4.4% |  |
| I smoke cigarettes every day | 332 | 14.0% | 351 | 12.9% |  |
| **Marital status** |  |  |  |  |  |
| Never married | 470 | 20.2% | 454 | 16.9% | <0.001 |
| Married or civil partner | 1,533 | 65.9% | 1,760 | 65.7% |  |
| Divorced, widowed, separated, former civil partner | 322 | 13.9% | 466 | 17.4% |  |
| **Highest academic qualification** |  |  |  |  |  |
| No formal qualifications | 693 | 29.7% | 620 | 23.0% | <0.001 |
| Up to A levels or diploma (typically attained at age 18) | 1,003 | 43.0% | 1,310 | 48.5% |  |
| Degree or higher | 636 | 27.3% | 771 | 28.5% |  |
| **Disability classification EU-SILC** |  |  |  |  |  |
| No EU-SILC long-standing health condition | 2,068 | 87.4% | 2,240 | 82.1% | <0.01 |
| EU-SILC classification to some extent | 199 | 8.4% | 334 | 12.2% |  |
| EU-SILC classification severely hampered | 100 | 4.2% | 154 | 5.7% |  |
| **Body mass index** (kg/m^2^) | 28.6 | ±4.8 | 27.9 | ±6.0 | <0.001 |
| **Sleep** (hours/day) | 5.87 | ±1.1 | 6.39 | ±4.8 | <0.001 |
| **Total activity** (minutes/day) | 154.4 | ±53.7 | 149.7 | ±50.2 | <0.005 |

| **Supplementary Table 2.** Associations between DFA score and each raw cognitive domain score at age 46 (β (95% confidence interval); change in cognitive score per 0.1 increase in DFA coefficient, n= 5 097) | | | | | | |
| --- | --- | --- | --- | --- | --- | --- |
|  | **Model i: unadjusted** | | **Model ii: adjusted for** education, self-reported health, disability, BMI, smoking, alcohol and sleep | | **Model iii: adjusted for** education, self-reported health, disability, BMI, smoking, alcohol, sleep and total PA time | |
| **FEMALES (n=2729)** | **B coefficient (95% CI)** | **p-value** | **B coefficient (95% CI)** | **p-value** | **B coefficient (95% CI)** | **p-value** |
| Verbal fluency (animals named) | 0.32 (-0.04, 0.67) | 0.09 | 0.16 (-0.19, 0.51) | 0.36 | 0.13 (-0.22, 0.48) | 0.47 |
| Processing speed (words searched) | 0.10 (-4.86, 5.05) | 0.97 | -0.31 (-5.29, 4.66) | 0.90 | -0.99 (-5.97, 4.00) | 0.70 |
| Immediate recall (words recalled) | 0.01 (-0.07, 0.10) | 0.75 | -0.03 (-0.12, 0.05) | 0.43 | -0.03 (-0.11, 0.05) | 0.48 |
| Delayed recall (words recalled) | 0.01 (-0.09, 0.12) | 0.80 | -0.05 (-0.15, 0.06) | 0.39 | -0.03 (-0.14, 0.07) | 0.53 |
| **MALES (n=2368)** | |  |  |  |  |  |
| Verbal fluency (animals named) | 0.65 (0.27, 1.02) | <0.001 | 0.43 (0.06, 0.80) | 0.02 | 0.43 (0.06, 0.81) | 0.02 |
| Processing speed (words searched) | 6.10 (1.20, 11.0) | 0.02 | 3.43 (-1.48, 8.36) | 0.17 | 3.69 (-1.26, 8.65) | 0.14 |
| Immediate recall (words recalled) | 0.11 (0.03, 0.20) | 0.01 | 0.03 (-0.05, 0.11) | 0.46 | 0.04 (-0.05, 0.12) | 0.37 |
| Delayed recall (words recalled) | 0.09 (-0.01, 0.20) | 0.09 | 0.00 (-0.10, 0.11) | 0.98 | 0.02 (-0.08, 0.13) | 0.65 |
| ^a ‑^Missing covariate date imputed using multiple imputation by chained equations | | | | | | |
